# Investigating the Associations of ADHD Symptoms, Impulsivity, Physical Exercise, and Problematic Pornography Use in a Chinese Sample

**DOI:** 10.3390/ijerph192215221

**Published:** 2022-11-18

**Authors:** Ying Zhang, Lijun Chen, Xiaoliu Jiang, Beáta Bőthe

**Affiliations:** 1 Department of Physical Education, Fuzhou University, Fuzhou 350108, China; 2 Department of Psychology, School of Humanities and Social Sciences, Fuzhou University, Fuzhou 350108, China; 3 Département de Psychologie, Université du Québec à Trois-Rivières, Trois-Rivières, QC G8Z 4M3, Canada; 4 Département de Psychologie, Université de Montréal, Montréal, QC H3T 1J4, Canada

**Keywords:** attention-deficit/hyperactivity disorder (ADHD), problematic pornography use (PPU), impulsivity, physical exercise, latent moderated structural equations (LMS)

## Abstract

Investigating the relationship between attention-deficit/hyperactivity disorder (ADHD) symptoms and impulsivity will benefit our understanding of the concept of problematic pornography use (PPU), and revealing predisposing and malleable moderators of PPU will be beneficial for its prevention and intervention. The current study not only aimed to observe these relationships, but also explored the potential moderating role of physical exercise in the general population. A total of 600 Chinese adults (*M*_age_ = 32.31, *SD*_age_ = 12.40, 39.8% women) were recruited and completed an online survey. The results showed that participants with regular exercise scored lower than those without exercise on ADHD, impulsivity, and PPU (all *p*s < 0.001). Using latent moderated structural equations (LMS), the findings revealed that the relationship between ADHD symptoms and PPU was mediated by impulsivity, and physical exercise moderated this relationship (B = −0.14, *p* = 0.048). Specifically, when individuals’ physical exercise was higher than 0.84 standard deviations above the mean, the positive predictive effect of impulsivity on PPU was not significant. These findings indicate the important role of impulsivity in the relationship between ADHD and PPU, and physical exercise could be a meaningful component of interventions among individuals experiencing PPU.

## 1. Introduction

The availability of the internet has provided people with unprecedented access to sexually explicit materials [1,2,3], even in sexually conservative countries [4,5]. A large body of evidence shows that a significant percentage of people (5–14%) who use pornography may encounter problematic pornography use (PPU) [6,7]. PPU, which is regarded as a subcategory of compulsive sexual behavior disorder (CSBD) in the 11th edition of the International Classification of Diseases (ICD-11), is characterized by persistent, excessive, or compulsive use of pornographic content and intensive engagement in it despite distress and adverse consequences [8,9,10,11,12,13]. However, such a classification has been questioned [14]. Some researchers considered the conceptualization of PPU as an impulse control disorder [15], while others believed that it should be a potentially addictive behavior [16]. An improved understanding of the possible association of impulsivity and PPU may help with respect to diagnosing PPU; moreover, deeply comprehending the working mechanisms of impulsivity may be beneficial for developing improved interventions [17,18].

Impulsivity is a multidimensional construct (e.g., sensation seeking, negative urgency, positive urgency, lack of perseverance, lack of premeditation), referring to the tendency to act prematurely and without foresight, and is agreed to be a core symptom of attention-deficit/hyperactivity disorder (ADHD) [19,20]. Therefore, ADHD is a non-negligible potential comorbidity when examining the role of impulsivity. ADHD is a neuropsychological disorder that is manifested by poor attention, hyperactive and impulsive behaviors [21,22], beginning in childhood and often persisting through adulthood. Previous studies have revealed solid association between ADHD and hypersexuality/CSBD [23,24]. In line with the potential relationship patterns of ADHD and hypersexuality/CSBD, ADHD should be considered one of the predisposing factors for PPU, as PPU may represent a prominent manifestation of hypersexuality/CSBD [17,18,19]. Moreover, in the few studies that have explored ADHD and PPU directly, ADHD symptoms positively and moderately predicted PPU [18]; ADHD and attachment difficulties contributed significantly to the variance of PPU, and explained 34% of the variance in PPU [25]. However, these studies only reported the extent to which ADHD symptoms were associated with PPU and did not explore the underlying mechanisms, limiting our understanding.

As impulsivity is a prominent manifestation of ADHD, several studies showed that individuals with ADHD demonstrated elevated levels of impulsivity as measured by a variety of tasks [26] and self-report measures as well [27], ADHD may have effect on PPU through impulsivity. Impulsivity has been identified as a shared vulnerability factor of specific internet use disorder including PPU [28]. Previous studies have also showed moderate, positive associations between impulsivity and problematic sexual behaviors, such as hypersexuality/CSBD [29,30] and PPU [31]. For instance, young men with problematic internet use reported higher level of impulsivity [32]; impulsivity was associated with CSBD in a predominantly female sample [31]; lack of perseverance (a dimension of impulsivity) was also associated with PPU among the youth [33]; and people with higher levels of sensation seeking (another dimension of impulsivity) used pornography more intensively: manifested by either an increased amount of time spent on pornography or the development of PPU [4]. However, there are contradictory results reported by Bőthe et al. [17,18], showing that impulsivity was weakly related to PPU. The inconsistent results suggest that the relationship between impulsivity and PPU may depend on or interact with other variables.

**Figure 1:**
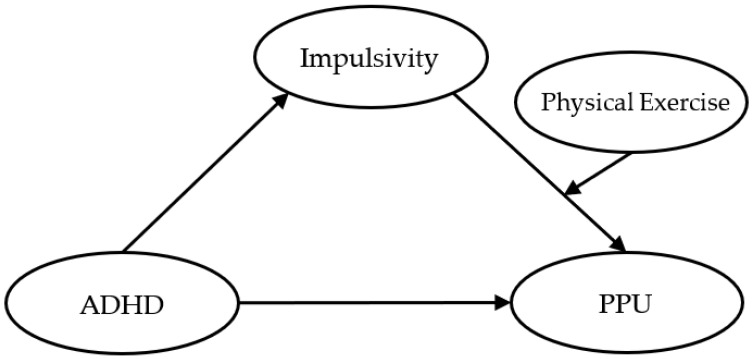
Hypothesized moderated mediation model of the associations between attention-deficit/hyperactivity disorder (ADHD), impulsivity, problematic pornography use (PPU), and physical exercise.

Since physical exercise is frequently used as a non-pharmacological complementary treatment recommendation for impulsivity-related psychopathologies [21,34,35], physical exercise may be a malleable moderator of this association between impulsivity and PPU. If the regulatory effect of physical exercise is identified, it will aid in finding an efficient and cost-effective intervention mode for PPU. Seminal work has revealed that physical exercise upregulated chronic and acute synthesis and release of several monoamines (e.g., serotonin, dopamine) [36], and high impulsivity was accompanied by low circulating and central of serotonin and its precursor, tryptophan [37,38]. This biological complementation is manifested in the observable phenomenon that physical activities, including physical exercise, can strengthen inhibitory control and reducing impulsivity [39]. Pilot studies have shown that physical exercise might be related to lower levels of impulsivity among general population [34], among children [21], adolescents and adults with ADHD [21,40,41]. Hypothetically, individuals regularly doing physical exercise might have higher levels of impulse control, so that their impulsivity may not be related to their PPU, whereas individuals lacking physical exercise might not inhibit their impulses as effectively, resulting in higher levels of PPU. Thus, regular physical exercise may be a moderator between impulsivity and PPU. Yet, it is important to note that the association between physical exercise and impulsivity may not always be negative as some studies have shown that excessive exercise was associated with high impulsivity [42]. Therefore, it is necessary to explore what levels of exercise can result in a weaker association between impulsivity and PPU (Figure 1).

## 2. Materials and Methods

### 2.1. Participants and Procedure

Data collection occurred on a popular Chinese survey website (www.wjx.cn (accessed on 2 December 2021)) in December 2021. The study was advertised as a research project about problematic pornography use, including impulsivity and ADHD symptoms. It took approximately 8 min to complete the survey. Participants were informed about the aims of the survey and would receive CNY 10 in compensation after completing it. Informed consent was obtained from the participants before data collection, and participants were assured of their anonymity. Eligible participants had to be 18 years old or older and had used pornography in the past six months. To prevent potential biases rooting in individual heterogeneity [43], a clear definition of pornography was provided to the participants before the pornography-related measures were presented [44,45,46]. In this study, pornography was defined as material that (i) creates or elicits sexual feelings or thoughts and (ii) contains explicit exposure or descriptions of sexual acts involving the genitals, such as vaginal or anal intercourse, oral sex, or masturbation [44,45].

In terms of sample size, there is no agreement on the sample size requirements for structural equation model (SEM). According to Wolf et al. [47], it is not easy to develop generalized guidelines regarding sample size requirements for SEM models despite the various rules of thumb that have been advanced. Some studies mention that SEM requires a “large” sample(e.g., 300 participants) [48,49]. In this study, we attempted to calculate the required sample size using a tool developed by Dr. Daniel S. Soper [50] based on Westland’s research [51]. After entering the number of observed and latent variables into the tool and setting the anticipated effect size to medium (0.3), as well as the desired probability and statistical power levels to 0.05 [52], the calculator returned the minimum sample size required to detect the specified effect as 207. Considering that structural equation model (SEM) requires a large sample, we collected as much data as possible.

A total of 803 participants willing to provide informed consent were recruited using this online sampling method. Before the analyses, the data were screened and participants were removed for the following reasons: (i) were underaged (24 individuals); (ii) either had failed any of the three attention test questions (e.g., Please select “often” in this item to indicate that you are filling out the survey carefully.) or provided contradicting responses (e.g., the actual age was younger than the age of first exposure to pornography) (66 individuals); (iii) had not used pornography in the past six months (113 individuals). Therefore, a total of 600 adult respondents (women = 239, 39.83%) from 29 of the 34 provinces/regions in China were included in the study for further analyses who were between 18–68 years old (*M*_age_ = 32.31, *SD*_age_ = 12.40, more details see Table 1).

### 2.2. Measures

#### 2.2.1. Short UPPS-P Impulsivity Scale (UPPS-P)

The short UPPS-P is a 20-item scale developed by Billieux et al. that evaluates five different impulsivity facets (four items per each dimension), including negative urgency (e.g., When I am upset I often act without thinking), positive urgency (e.g., When overjoyed, I feel like I cannot stop myself from going overboard), lack of premeditation (e.g., I usually think carefully before doing anything), lack of perseverance (e.g., I generally like to see things through to the end), and sensation seeking (e.g., I sometimes like doing things that are a bit frightening) [53]. All items are scored on a Likert scale from 1= *agree strongly* to 4 *= disagree strongly*, with the three aspects of negative urgency, positive urgency, and sensation seeking scored in reverse. Higher values indicate more impulsive behavior. The Cronbach’s alpha of the entire scale was 0.84 in this study (0.79–0.82 across dimensions), suggesting good internal consistency.

#### 2.2.2. The Six-Question Adult ADHD Self-Report Scale (ASRS)

The Six-Question ASRS is the short form of the ASRS based on the DSM-IV symptom list, developed by the workgroup on adult ADHD in collaboration with the World Health Organization [54]. Each question asked how often a symptom occurred over the past 6 months on a Likert scale from 0 = *never* to 4 = *very often*. The total score is calculated by summing the values of all items. The higher the score, the more pronounced the symptoms are. The Cronbach’s alpha of the entire scale was 0.71 in this study.

#### 2.2.3. The Brief Pornography Screen (BPS)

The BPS is a short screening tool focusing on measuring the lack of self-control and overuse of pornography to identify individuals who might be at risk of PPU (e.g., You have attempted to “cut back” or stop using pornography, but were unsuccessful) [55]. This five-item scale uses a Likert scale from 0 = *never* to 2 = *very often*. Higher scores (range = 0–10) indicate higher levels of impaired control regarding pornography use, and the cut-off is 4 points. The Cronbach’s alpha of the entire scale was 0.85 in this study.

#### 2.2.4. Physical Exercise

We assessed participants’ physical exercise habits with two items developed for this study: “How often do you engage in moderate or high intensity (e.g., cycling, running, jumping rope) exercise per month?” Responses included: 1 = *less than once a month*, 2 = *2 to 3 times a month*, 3 = *1 to 2 times a week*, 4 = *3 to 5 times a week*, 5 = *about once a day*. “How long do these sessions last each time?” Responses included: 1 = *less than 9 min*, 2 = *10 to 19 min*, 3 = *20 to 29 min*, 4 = *30 to 59 min*, 5 = *one hour or more*. The Cronbach’s alpha of the two items was 0.67 in this study. According to the *Guidelines of Healthy China Initiative 2019–2030*, regular physical activity was defined as exercising more than three times per week of moderate-intensity physical activity lasting at least 30 min [56]. This definition was also applied in other studies [57,58]. Basing on this criterion, if the participants respond “4” and above to the two items, it means that he/she is a person with regular exercise, and we can divide the participants into regular exercise group and no exercise group.

### 2.3. Analysis

Statistical analyses were conducted using SPSS 22.0 (IBM, Armonk, NY, USA) and Mplus 8.3 (Muthén & Muthén, Los Angeles, CA, USA). Descriptive statistics were conducted to determine the means, standard deviations (*SD*s), and internal consistencies for each scale using SPSS. The demographics of the regular exercise group and no exercise groups were compared using the chi-square test for categorical variables and the independent-sample *t*-test for continuous variables. After controlling for the effects of gender and age, partial correlations were used to examine the strengths of relationships between the main research variables including ADHD, impulsivity, PPU, and physical exercise habits.

Then, we examined a mediation model and a moderated mediation model with latent moderated structural equations (LMS) in Mplus 8.3. LMS represents an extension of ordinary SEM as they explicitly take into account the nonnormality caused by the latent non-linear interaction terms [59]. Simulation studies have shown that LMS provide efficient parameter estimators and a reliable model difference test, with no sign of bias in standard errors [59,60,61]. The LMS is statistically rigorous and robust to nonnormality when compared to conventional approaches (e.g., moderated multiple regression, product indicator approach) for estimating latent variable interactions [59,62].

We followed the four-step procedure recommended by Cheung et al. to estimate moderating effects of latent variables with LMS [61]. The four-step procedure starts by evaluating measurement quality using confirmatory factor analysis (CFA) (step 1: Assessment of the measurement model, model 1). Next, we established baseline fit indices with the model without latent interactions (step 2: SEM without latent interaction, model 2). Then, the latent interaction (i.e., impulsivity and physical exercise habits) was added to the LMS model so that the index of moderated mediation could be calculated (step 3: Latent variable moderated mediation, model 3). As Mplus does not provide conventional fit indices for evaluating the overall model fit when estimating an LMS model, we performed a chi-square difference test based on the log likelihood values and scaling correction factor [63] estimated from Model 2 and Model 3 using the calculator provided by Cheung et al. [61]. Model 3 would be deemed to better fit the data than Model 2 if the log-likelihood ratio test produced a significant value and the latent interactions could significantly predict PPU. Finally, we conducted simple slope tests and tested the interaction effects (Step 4: Interpreting the interaction effect, model 4).

The values of the comparative fit index (CFI; acceptable > 0.90), root mean square error of approximation (RMSEA; acceptable < 0.08), and standardized root mean square residual (SRMR; acceptable < 0.08) were used to evaluate the model fit [64]. The bootstrapping (*n* = 2000) technique and its 95% confidence interval (CI) were employed to determine the significance of the (moderated) mediation effect. If the 95% CI does not include 0, then a significant (moderated) mediation effect is present.

### 2.4. Ethics Approval

The study was approved by the local ethics committee of Fuzhou University and was conducted following the ethical standards required for conducting human research in accordance with the fundamental principles in the Declaration of Helsinki. The datasets in this study were collected and analyzed anonymously. Participants were informed about the procedure, the purpose of the study, the investigation responsibility unit, the anonymity of their data, and the approximate length of the survey. As in many other studies [65,66], to obtain online informed consent, all participants were asked to check a box within the online survey. This method was used to protect the participants’ anonymity.

## 3. Results

### 3.1. Preliminary Analysis: Descriptive Statistics and Correlations

Table 1 shows the samples’ sociodemographic characteristics. To better investigate the potential effect of sociodemographic factors, we divided participants into two groups according to the definition of exercise habits: the regular exercise group (i.e., both questions received a score of 4 or higher) and the no exercise group (i.e., other participants). The chi-square tests and the independent-sample *t*-tests’ findings revealed no significant differences between the two groups except for the gender ratio, as more men belonged to the regular exercise group. Table 2 includes descriptive data of impulsivity, ADHD, PPU, and physical exercise habits. Given gender differences in PPU and exercise habits (*p*s < 0.05), gender was added as a covariate in the following primary analyses. Men did more physical exercise than women and scored higher on PPU. The regular physical exercise group showed lower scores on ADHD, impulsivity and PPU than the no regular physical activity group.

In Table 3, Pearson’s partial correlation coefficients are shown between ADHD symptoms, PPU, physical exercise, and impulsivity and its dimensions. PPU was moderately and positively associated with ADHD and impulsivity, as well as ADHD and impulsivity with each other, while physical exercise showed negative but weak associations with ADHD, impulsivity, and PPU. These preliminary findings provided support for testing the main hypothesized model.

### 3.2. Primary Analyses to Test the Moderated Mediation Model: ADHD Symptoms as Independent Variable, Impulsivity as Mediator, Physical Exercise as Moderator, PPU as Dependent Variable

#### 3.2.1. Step 1: Assessment of the Measurement Models (Model 1)

The measurement model consisted of four inter-correlated latent factors. Dimensions were used as measured variables for impulsivity (five indicators) and items were used as the measured variables for ADHD (six indicators), physical exercise (two indicators) and PPU (five indicators). Results from the confirmatory factor analysis demonstrated an acceptable fit to the data, χ^2^ (129) = 467.203, *p* < 0.001, CFI = 0.90, RMSEA = 0.07 (90% CI = 0.060, 0.073), SRMR = 0.07. Unstandardized factor loadings of all indicators were statistically significant and standardized factor loadings were greater than the threshold value of 0.40 [67] except for one dimension of impulsivity (i.e., lack of premeditation, factor loading = 0.10, *p* = 0.059). Considering that the fit of the model was acceptable, this dimension of impulsivity was retained in this study. The correlation coefficients among all latent variables ranged from −0.26 to 0.61, indicating that they were four distinct variables because the absolute value of all correlation coefficients was significantly lower than 1 [61]. These results provided evidence for adequate construct validity, as well as discriminant validity.

#### 3.2.2. Step 2: SEM without Latent Interaction (Model 2)

Following the assessment of the measurement model, an SEM without the latent interaction term (Model 2) was estimated. We only estimated the main effects of impulsivity, ADHD, and physical exercise on PPU. Model 2 had an acceptable fit to the data: χ^2^ (147) = 420.77, *p* < 0.001, CFI = 0.90, RMSEA = 0.06, 90% CI [0.058, 0.070], SRMR = 0.07. The results showed that the main effect of impulsivity (β = 0.36, B = 0.30, *p* < 0.001), ADHD (β = 0.17, B = 0.17, *p* = 0.015) and gender (β = −0.21, B = −0.23, *p* < 0.001) on PPU were statistically significant. All independent variables together accounted for 29.0% of the variance in PPU. As the model without the latent interaction fit the data well, we could proceed to Step 3.

#### 3.2.3. Step 3: LMS Model Estimation (Model 3)

**Figure 2:**
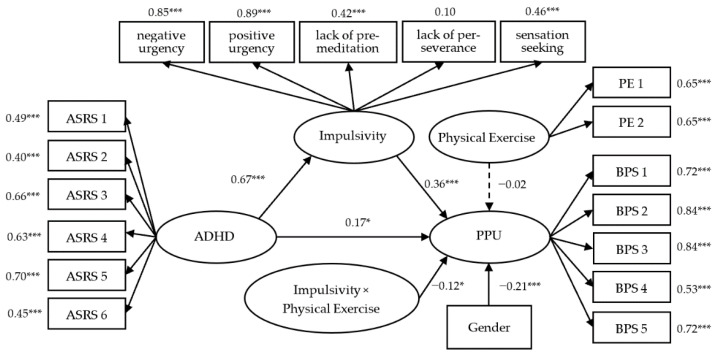
The latent moderated mediation model (Model 3). Standardized coefficients are reported. * *p* < 0.05, *** *p* < 0.001.

Following the LMS procedure, the latent interaction (i.e., impulsivity and physical exercise) was added to Model 3. To evaluate the model fit of this LMS model, we conducted a chi-square difference test based on the log likelihood values and scaling correction factors estimated from model 3 (L1 = −11,853.755, c1 = 1.072) and model 2 (L0 = −11,855.931, c0 = 1.079). The results indicated that Model 3 showed a significant improvement in model fit (Satorra–Bentler Scaled χ^2^ (1) = 7.05, *p* < 0.01), and physical exercise significantly moderated the relationship between impulsivity and PPU (β = −0.12, B = −0.14, *p* = 0.048, 95% CI [−0.33, −0.02]). The estimated index of moderated mediation was −0.10, 95% CI [−0. 25, −0.01] and the indirect effect of mediation was 0.22, 95% CI [0.13, 0.33]. All the independent variables accounted for 30.1% of the explained variance in PPU (Figure 2).

#### 3.2.4. Step 4: Interpreting the Interaction Effect (Model 4)

**Figure 3:**
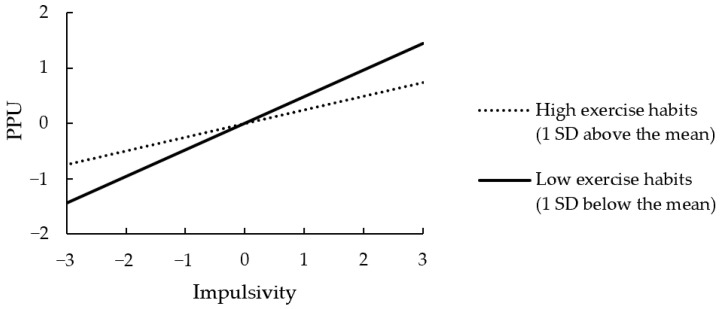
The standardized simple slope analysis for the moderating effect of exercise habits.

**Figure 4:**
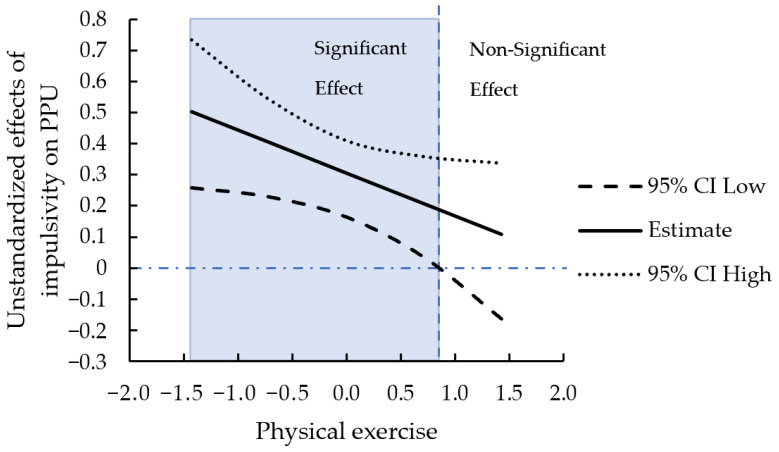
Unstandardized effects of impulsivity on PPU conditional on physical exercise.

To better understand the moderation effect, we conducted a simple slope test using bootstrapping to examine the statistical significance of the effect of impulsivity on PPU, with given conditional values of exercise habits (*M* ± 1 *SD*) (Figure 3). In both conditions with low and high exercise, impulsivity was positively and significantly related to PPU (B = 0.40, *p* < 0.001, 95% CI [0.23, 0.54] and B = 0.21, *p* = 0.014, 95% CI [0.03, 0.36]), with having a steeper slope in the case of low exercise. We also plotted the interaction effect with the Johnson–Neyman technique [68] provided by Cheung et al. [61]. Figure 4 demonstrates a boundary condition for the effect of impulsivity on PPU. Impulsivity had no statistically significant positive effect on PPU when the level of physical exercise was higher than 0.84 standard deviations above the mean (3.43 ± 0.94). This level was close to reaching the criteria for regular exercise (responding “4” to the two items of physical exercise).

## 4. Discussion

Exploring potential predisposing factors of PPU will contribute to better understanding its development and maintenance. Among these characteristics, ADHD and impulsivity were deemed important in prior studies, but no closer look was taken on these variables considering them concurrently. The present study not only revealed a positive and moderate association among ADHD, impulsivity and PPU, but also showed that impulsivity worked as a mediator in this association, that is, individuals with higher levels of ADHD may experience higher levels of impulsivity, which, in turn, may result in higher levels of PPU. Additionally, the predictive level of impulsivity on PPU depended on the individual’s amount of physical exercise. More precisely, for those individuals who exercised regularly, the predictive effect of impulsivity on PPU seemed to disappear. These results provide information for developing efficient and cost-effective intervention strategies for PPU.

This study found a moderate and positive association between ADHD and PPU, which was slightly higher than in recent previous studies [18]. As suggested, pornography use might be a negative reinforcement for individuals with ADHD, since they may experience peer rejection, problems in romantic relationships, and employment difficulties, which may make them vulnerable to use pornography as a way of ‘escaping’ or ‘avoiding’ emotional discomfort [69]. Moreover, one of the potential reasons for the stronger association in this study compared to previous work [18] might relate to the different cultural context where the samples were recruited. Most studies reported that Chinese people’s attitude toward sex is more conservative [70], and they are more likely to experience guilty and conflicted as using pornography [71]. Therefore, when the participants with high levels of ADHD symptoms used pornography to cope with their stressful situations, they may be inclined to think that they have used “too much”, and then scored higher in BPS. There is evidence that frequency of use was more strongly associated with PPU in conservative contexts than in permissive contexts [70].

In addition to the direct effect of ADHD symptoms on PPU, the mediation of impulsivity in the association between ADHD and PPU implied that impulsivity might be a possible mechanism explaining this association. The mediating role of impulsivity might root in the fact that urgency and sensation seeking (the two major dimensions of impulsivity) are common features of ADHD [20,72], which was supported by the higher correlation coefficients for ADHD and PPU with these two dimensions (see Table 2). Firstly, ADHD can be a consequence of reward deficiency [73], with altered reinforcer sensitivity [74]. Individuals with ADHD demonstrated more impulsively in delay-discounting tasks, preferring smaller but more immediate rewards to larger but more delayed rewards (performance of the urgency) [75], and viewing online pornographic materials is pleasurable or rewarding and easily accessible in a timely manner. Therefore, individuals with ADHD symptoms might be more likely to be attracted to pornographic materials than the general population. Additionally, sensation seeking refers to the tendency to seek various novel, complex and arousing sensory stimulations, and openness toward new experiences [76]. There is evidence that the prefrontal cortex (PFC) is hyperactive in individuals with ADHD [77,78], which is linked to attention deficits and the difficulty of staying focused for a prolonged time, and the sensation seeking observed in ADHD was interpreted as an autoregulatory attempt to create a stimulating environment in order to stabilize vigilance, which may explain the higher need for sexual desire, masturbation, or viewing pornography in individuals with ADHD [69].

According to the notions of the I-PACE (Interaction of Person-Affect-Cognition-Execution) model, impulsivity was identified as an important predisposing factor of PPU [79]; however, previous studies did not show a robust and consistent association between impulsivity and PPU. The present study revealed that the amount of physical exercise may explain this inconsistency. For those who had regular exercise habits, impulsivity might relate less strongly to PPU than among those who did not exercise regularly. There is substantial evidence that physical exercise reduces impulsivity. Adherence to exercise (60 min of moderate-to-vigorous physical activity per day) enhanced the treatment and prevention of impulsivity-related disorders [80]; physical activity improved attention and executive functions and reduced hyperactivity and impulsivity in children with ADHD [81]. Among adolescents, less frequent vigorous physical activity was associated with greater negative urgency and lack of perseverance [82], and the amount and intensity of physical exercise per week were negatively correlated with emotion-related impulsivity levels [83]. The effect of physical exercise on impulsivity is due to the fact that impulsivity has been tied to the monoamine pathway [84,85], and this pathway can be strongly modulated by physical exercise [86,87]. Recently, a further physiological study explored physical exercise interventions on impulsivity from a physiological perspective and reported that an eight-week high-intensity interval training was able to switch the kynurenine pathway from its neurotoxic branch to its neuroprotective one, compared to the active stretching group. This shift was associated with a decrease in impulsivity [35].

There were some limitations in the present study that needed to be addressed. Because the data was collected using anonymous self-report measures, the reliability of the results is dependent on the respondents’ honesty and accuracy in understanding the scale items. Due to the use of self-reported scales without clinical diagnosis, only the associations of ADHD symptoms, impulsivity, and PPU could be examined without establishing comorbidity rates. Future studies may apply various research methods to assess not only the self-reported severity of ADHD symptoms, impulsivity, and PPU but also clinical diagnosis. Given that impulsivity is a multi-dimensional and multi-faceted construct and different dimensions may play different roles in PPU [79], more research is needed on the dimensions/facets of impulsivity. Furthermore, as there are differences in ADHD sub-type comorbidity [88], additional research is needed to determine whether impulsivity and PPU are comorbidities in all ADHD sub-types. Although the sample was recruited throughout China, it was not representative in nature and only examined those who used the Internet. In addition, participation in the research was voluntary; thus, those individuals who were not interested in the topic of the survey might have declined to participate. Finally, causality cannot be inferred from this cross-sectional study and the biological mechanisms of ADHD, impulsivity, and PPU are unknown. More scientific studies are needed to determine whether they share common physiological origins.

Impulsivity did contribute as importantly and directly to PPU in this study as previous studies suggested. This result has conceptual and research implications: supporting that PPU is a sub-type of CSBD and that it is reasonable to be included under the classification of impulse control disorders in the ICD-11. In line with the results of previous studies, ADHD may contribute to individuals’ PPU [18,25]. Thus, in the case of individuals seeking treatment for PPU in clinical practice, clinicians and therapists should also assess ADHD symptoms as PPU may be a symptom of the “real” problem: ADHD. Furthermore, physical exercise was found to be significantly associated with impulsivity and PPU and the association between impulsivity and PPU was moderated by physical exercise (i.e., the association was weaker at higher levels of physical exercise). Our findings indicate that regular exercise may help individuals reduce their impulsivity levels and PPU and can be a therapeutic approach to prevent the development of impulse-related disorders. However, it is critical to exercise in moderation to avoid the development of exercise dependence/addiction [89]. Since exercise addiction showed a higher neurotic personality trait and exhibited an impaired reward system [90], it may worsen the severity of symptoms of impulsivity and PPU.

## 5. Conclusions

The findings of the present study showed that impulsivity was a mediator between ADHD and PPU, physical exercise moderated the relationship between ADHD and PPU. Thus individuals with PPU should also be screened for ADHD in the diagnostic process and exercise might help individuals reducing their PPU.

## Figures and Tables

**Table 1:** Sociodemographic characteristics of the sample.

Characteristic		Total (*n* = 600)	No Exercise Group (*n* = 373)	Regular Exercise Group ^1^ (*n* = 227)	χ^2^/*t*	*p*	*Cohen’s d* */Cramer’s V*
Gender ratio (men/women) ^2^		1.51	1.30	1.95	5.33	0.021	0.09
Age of first exposure to pornography		16.39 ± 4.68	16.18 ± 4.82	16.75 ± 4.42	−1.47	0.143	0.12
Age	Mean ± SD	32.31 ± 12.40	31.99 ± 11.91	32.84 ± 13.19	−0.82	0.413	0.07
	Range	18–68	18–52	18–68	-	-	-
Sexual orientation	Homosexual	78 (13.00%)	47 (12.60%)	31 (13.66%)	5.80	0.122	0.09
	Heterosexual	469 (78.17%)	289 (77.48%)	180 (79.30%)			
	Bisexual	51 (8.50%)	37 (9.92%)	14 (6.17%)			
	Asexual	2 (0.33%)	0 (0.00%)	2 (0.88%)			
Relationship status	Single	244 (40.67%)	150 (40.21%)	94 (41.41%)	0.08	0.773	0.01
	Partnered	356 (59.33%)	223 (59.79%)	133 (58.59%)			
Educational level	Primary	3 (0.50%)	3 (0.80%)	0 (0.00%)	2.88	0.236	0.07
	Secondary	51 (8.50%)	35 (9.38%)	16 (7.05%)			
	Tertiary	546 (91.00%)	335 (89.81%)	211 (92.95%)			
Work status	No job	175 (29.17%)	111 (29.76%)	64 (28.19%)	3.49	0.322	0.08
	Full time job	355 (59.17%)	213 (57.10%)	142 (62.56%)			
	Part time job	58 (9.67%)	42 (11.26%)	16 (7.05%)			
	Odd job	12 (2.00%)	7 (1.88%)	5 (2.20%)			

Note. ^1^ Physical exercise habits were assessed by the criterion exercising more than three times per week of moderate-intensity physical activity lasting at least 30 min. ^2^ Gender (0 = men, 1 = women).

**Table 2:** Descriptive statistics of study variables.

Variables	Total (*n* = 600)	*M (SD, Skewness, Kurtosis)* ^7^		*t* ^5^	*Cohen’s d*	*M (SD, Skewness, Kurtosis)*		*t* ^6^	*Cohen’s d*
		Men (*n* = 361)	Women (*n* = 239)			No Regular Exercise (*n* = 373)	Regular Exercises ^a^ (*n* = 227)		
Impulsivity ^1^	2.33 (0.39, 0.03, −0.06)	2.34 (0.39, 0.15, 0.04)	2.31 (0.39, −0.15, −0.25)	0.70	0.07	2.39 (0.39, 0.03, −0.02)	2.22 (0.38, 0.04, −0.11)	5.29 ***	0.44
I1 negative urgency	2.46 (0.71, 0.05, −0.38)	2.47 (0.73, 0.02, −0.38)	2.44 (0.69, 0.10, −0.38)	0.49	0.04	2.57 (0.69, 0.02, −0.41)	2.27 (0.70, 0.15, −0.27)	5.00 ***	0.43
I2 positive urgency	2.56 (0.66, 0.10, −0.42)	2.56 (0.65, 0.27, −0.35)	2.56 (0.68, 0.27, −0.50)	0.01	<0.001	2.67 (0.64, 0.18, −0.45)	2.37 (0.66, 0.54, −0.04)	5.49 ***	0.46
I3 lack of premeditation	1.91 (0.53, 0.30, 0.02)	1.89 (0.54, 0.33, 0.05)	1.92 (0.52, −0.26, −0.01)	−0.65	0.06	1.96 (0.52, 0.26, −0.12)	1.82 (0.55, 0.42, 0.33)	3.01 **	0.26
I4 lack of perseverance	1.90 (0.57, 0.23, −0.46)	1.93 (0.59, 0.22, −0.48)	1.86 (0.54, 0.26, −0.48)	1.42	0.12	1.93 (0.58, 0.30, −0.45)	1.85 (0.57, 0.15, −0.54)	1.72	0.14
I5 sensation seeking	2.81 (0.62, −0.19, −0.08)	2.83 (0.62, −0.10, −0.04)	2.78 (0.63, −0.30, −0.15)	0.91	0.08	2.83 (0.62, −0.30, 0.01)	2.78 (0.63, −0.01, −0.14)	0.88	0.08
ADHD ^2^	1.79 (0.72, 0.30, 0.28)	1.82 (0.74, 0.20, 0.01)	1.76 (0.67, 0.48, 0.91)	1.05	0.09	1.87 (0.71, 0.44, 0.63)	1.67 (0.72, 0.13, −0.39)	3.18 **	0.28
PPU ^3^	0.95 (0.60, −0.01, −1.11)	1.05 (0.60, −0.22, −0.90)	0.80 (0.60, 0.38, −1.05)	4.99 ***	0.42	1.01 (0.59, −0.03, −1.02)	0.84 (0.62, 0.08, −1.28)	3.34 ***	0.28
Physical Exercise ^4^	3.43 (0.94, −0.60, −0.04)	3.50 (0.49, −0.63, −0.05)	3.32 (0.49, −0.60, 0.06)	2.22 *	0.37	2.88 (0.75, −0.87, 0.32)	4.33 (0.34, 0.54, −0.74)	5.29 ***	2.49

Note. ^a^ Regular physical exercise was assessed by the criterion exercising more than three times per week of moderate-intensity physical activity lasting at least 30 min; ^1^ Impulsivity was measured by the UPPS-P scale, range = 1–4; ^2^ ADHD was measured by the ASRS scale, range = 0–4; ^3^ PPU was measured by the BPS scale, range = 0–2; ^4^ Exercise habits were measured by two items developed for this study, range = 1–5; ^5^ Results of the independent-sample *t*-tests for gender; ^6^ Results of the independent-sample *t*-tests for exercise habits; ^7^ *M* = mean, *SD* = standard deviation (in parenthesis); * *p* < 0.05, ** *p* < 0.01, *** *p* < 0.001.

**Table 3:** Correlations between study variables.

Variables	Impulsivity	I1	I2	I3	I4	I5	ADHD	PPU
Impulsivity ^1^	-							
I1 negative urgency	0.85 ***	-						
I2 positive urgency	0.84 ***	0.75 ***	-					
I3 lack of premeditation	0.56 ***	0.29 ***	0.23 ***	-				
I4 lack of perseverance	0.34 ***	0.10	−0.01	0.58 ***	-			
I5 sensation seeking	0.45 ***	0.35 ***	0.47 ***	−0.20 ***	−0.41 ***	-		
ADHD ^2^	0.38 ***	0.36 ***	0.38 ***	0.05	−0.01	0.31 ***	-	
PPU ^3^	0.41 ***	0.39 ***	0.43 ***	0.03	−0.13 ***	0.44 ***	0.36 ***	-
Physical Exercise ^4^	−0.22 ***	−0.19 ***	−0.17 ***	−0.20 ***	−0.16 ***	0.03	−0.11 **	−0.14 ***

Note. The correlation coefficient is the partial correlation coefficient after controlling for age and gender; PPU = problematic pornography use, I = impulsivity; ^1^ Impulsivity was measured by the UPPS-P scale, range = 1–4; ^2^ ADHD was measured by the ASRS scale, range = 0–4; ^3^ PPU was measured by the BPS scale, range = 0–2; ^4^ Exercise habits were measured by two items developed for this study, range = 1–5; ** *p* < 0.01, *** *p* < 0.001.

## Data Availability

The data presented in this study can be requested from the corresponding author.
